# Does consumption of pearl millet cause goiter? A systematic review of existing evidence

**DOI:** 10.3389/fnut.2024.1323336

**Published:** 2024-03-07

**Authors:** Seetha Anitha, Shweta Upadhyay, Stefania Grando, Joanna Kane-Potaka

**Affiliations:** ^1^Nutrition Expert, Asia-Pacific Association of Agricultural Research Institutions, Bangkok, Thailand; ^2^Independent Consultant, Ascoli Piceno, Italy; ^3^Deputy Director General for Strategy, Engagement and Impact, International Rice Research Institute, Los Baños, Philippines

**Keywords:** pearl millet, goiter, thyroid, goitrogenic factors, C-glycosylflavones

## Abstract

Millets (defined here to also include sorghum) have been consumed in Asian and African countries for centuries, and have in recent years become increasingly popular in Western countries, especially because of their proven health and environmental benefits. Nevertheless, some concerns have been raised that their consumption can interfere with thyroid function and cause goiter. This systematic review aimed to investigate the link between millet consumption and goiter. We found nine papers that were relevant to this topic and included them in this review. Among nine papers eight were on pearl millet and one was on fonio millet. The findings of the review indicate that published literature on the association of pearl millet and increased goiter prevalence are not compelling and strong enough to assert that pearl millet consumed as part of a balanced diet can lead to goiter in the general population. To ensure appropriate factual messaging about millets, we need more scientific research to conclusively state whether millet consumption mediates goitrogenic effects.

## 1 Introduction

Millets are an integral part of traditional diets in Asian and African countries (1, 2). More than 90 million people in about 30 countries depend on millets for food and income (3). The high concentration of nutrients in millet grains makes them suitable for food and nutritional security (4). The energy value and protein and other macronutrient contents of millets are equal to, or in some instances, more than those in major cereals such as maize, wheat, and rice (5). Recent scientific evidence collated from a series of systematic reviews and meta-analyses shows that millets help in managing diabetes (6) and the lipid profile (7), reducing anemia (8) and calcium deficiency (9), and improving growth in children (10). Millets also exhibit anticancer, anti-inflammatory, and antifungal properties (11). In recent years, millet consumption has been slowly gaining prominence across the world owing to its multiple health benefits, among other advantages, especially its potential in the prevention and management of non-communicable diseases such as type 2 diabetes, hyperlipidemia, obesity, cardiovascular disease (CVD), and cancer.

Recognized for their resilience and adaptability, millets can withstand challenging growing conditions such as drought, diseases, insects, pests, and nutrient-depleted soils (12). Given the current focus on sustainable food production in the context of shifting climate patterns and water scarcity, there has been growing interest in millets in developing as well as developed regions of the globe (13). There is renewed focus on enhancing their production to reduce the over-reliance on commonly grown crops, promote more diverse diets, and move toward food security (14). Enhanced cultivation and consumption of millets are expected to play a pivotal role in attaining the Sustainable Developmental Goals (SDGs), especially SDG 2 (Zero Hunger), SDG 3 (Good Health and Well-being), SDG 12 (Responsible Consumption and Production) and SDG 1 (Climate Action). As millets can play an important role in attaining these SDGs, the United Nations General Assembly declared the year 2023 as the International Year of Millets (IYOM) (14, 15). Acknowledging the role of millets in responding to nutritional, agrarian, and climate challenges, the UN resolution on IYOM contemplates the “urgent need to raise awareness on the millets and to advocate for diversified, balanced and healthy diets through the increased sustainable production and consumption of millets.” The Government of India too has been promoting millet production as part of its National Food Security Mission (NFSM). Millet grains, which used to be referred to as ‘coarse cereals’ or ‘cereals of the poor’ or ‘orphan crops’, have been rebranded as ‘Nutri cereals’ (16).

Although the health and nutritional benefits of millets are well-demonstrated, concerns have been raised that millet consumption adversely affects the functioning of thyroid gland. The thyroid gland secretes thyroid hormones thyroxine (T4) and triiodothyronine (T3) that regulate many metabolic processes, including growth and brain development in children and energy expenditure (17). Iodine is an indispensable component of the thyroid hormones and deficiency of iodine adversely affects the synthesis of thyroid hormones. During prolonged iodine deficiency iodide uptake of thyroid is seriously hampered and blood level of T4 start dropping. This leads to increased TSH secretion. Under TSH stimulation thyroid gland undergoes hypertrophy and hyperplasia of follicular cells, enlarges in size and appears as a goiter (18). Pearl millet has been speculated to contain goitrogenic chemicals, which inhibit iodine utilization by thyroid gland, and cause thyroid disorders by reducing thyroid hormone production. Given that production of millets is being promoted as a sustainable alternative to tackle food insecurity, such concerns need to be investigated. So far, there has been no systematic review conducted to evaluate the association between millet consumption and goiter. This is the first such literature review. Its aim is to evaluate the available evidence on the influence of pearl millet consumption on goiter and its clinical implications relating to the thyroid gland.

## 2 Materials and methods

### 2.1 Search strategy

This systematic review was conducted searching for predetermined terminologies on search engines such as Google Scholar and PubMed based on the following main search string; (TSH OR thyrotropin OR triiodothyronine OR thyroxine OR “Iodide peroxidase” OR “TPO” OR thyroid* OR T4 OR T3) OR hypothyroid* AND (millet* OR isoflavones OR C-glycosyl flavones).

### 2.2 Eligibility criteria

The review encompassed human and animal studies (cross-sectional and experimental studies) published until June 2023. Any paper regardless of the year published was included in the review, considering there have been only very limited number of studies. The criteria for inclusion of papers were: (1) both clinical studies and cross-sectional studies conducted to test millet consumption and its association with goiter; and (2) animal, plant, *in vitro*, and/or human studies that tested for goitrogenic substances or similar substances in millets. Since the initial search yielded only a limited number of studies with focus on goiter as an outcome, the authors included related outcomes pertaining to our aim in order to gain a more comprehensive perspective. Accordingly, all population groups were included. However, all citations without full text, anonymous reports, narrative reviews, letters, editorials, commentaries, and conference papers were excluded, but their reference lists were manually screened to identify additional references. The reference lists of all the articles included in the review were screened. Publications not in English were excluded.

### 2.3 Selection of sources of evidence

Two independent reviewers (SA and SU) did the initial screening of articles, their titles and abstracts based on predefined inclusion and exclusion criteria. If the reviewers agreed on the appropriateness of an article, it was subjected to a full-text screening and then reviewed again independently by both reviewers. In case of a disagreement, a third reviewer was consulted. Duplicate entries were removed during the process. All the studies were evaluated for quality as well.

### 2.4 Data extraction

Data extraction for the review followed a standardized format, adapted from the Joanna Briggs Institute (JBI) data extraction format (19). The data extraction format included the primary author, publication year, study site, study design, sample size, and the main results. In the case of studies investigating multiple outcomes, only those outcomes that aligned with the eligibility criteria were summarized.

### 2.5 Collating, summarizing, and reporting results

A narrative approach was used to succinctly outline the main findings of the studies. The selected studies were grouped on the basis of their outcome such that the results described the type of evidence they had found on the goitrogenic effect of millets. Any gaps in knowledge were highlighted.

### 2.6 Ethics statement

Since this is a review of publicly available peer-reviewed literature, it was not necessary to undergo an ethical review process.

## 3 Results

**Figure 1:**
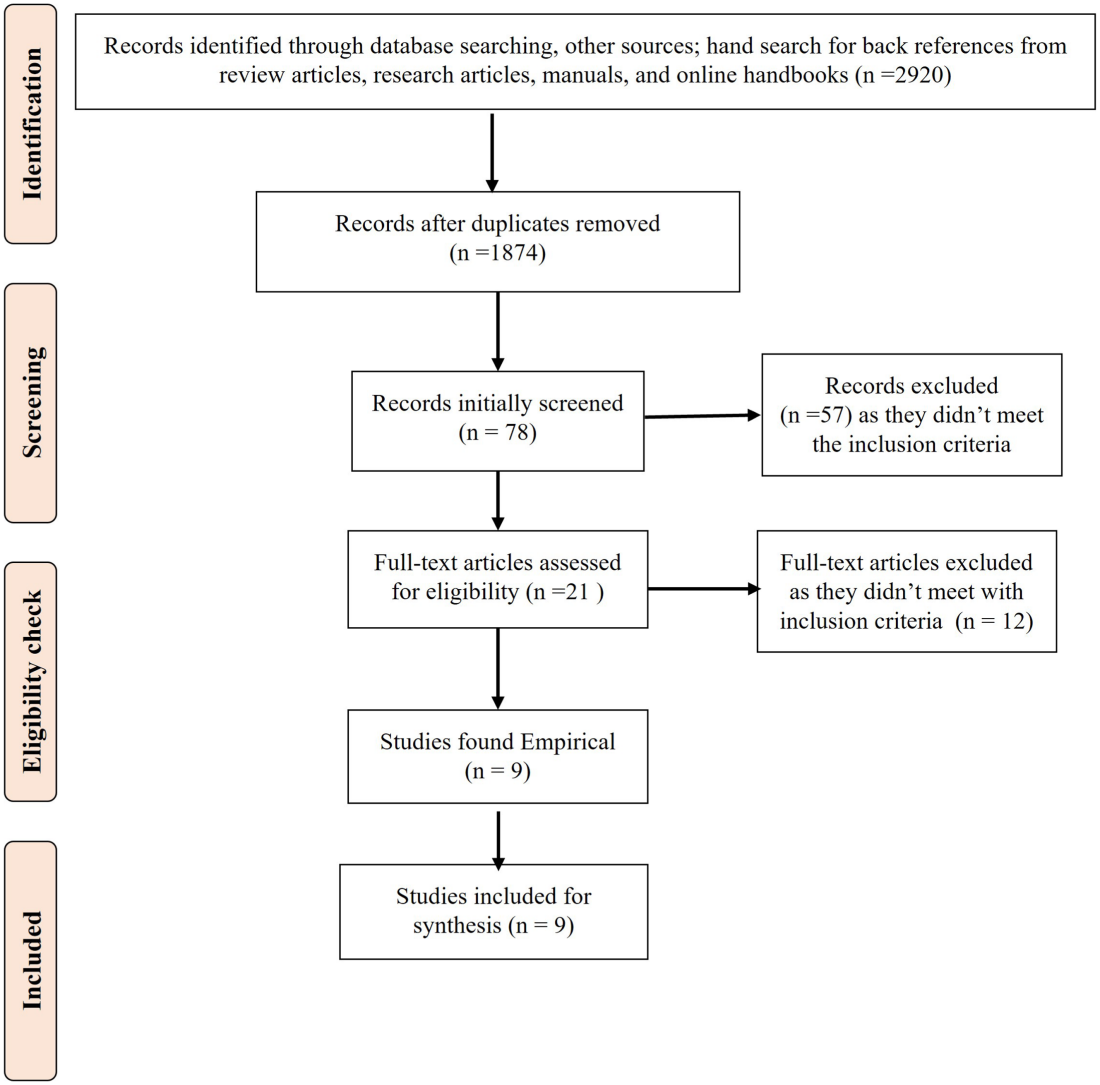
PRISMA flow diagram of the review process.

After a thorough screening, as illustrated in Figure 1, nine studies (20–28) were found eligible for inclusion in the review.

### 3.1 Study characteristics

Table 1 provides a description, without any order of preference, of the studies included in this systematic review. Six studies (22–24, 26–28) had an experimental design (four *in vivo* studies, one *in vitro* study and one that used both *in vivo* and *in vitro* methods); two were cross-sectional (20, 25); and one (21) used a mixed approach (combining cross-section survey and experimental analysis). All of them considered the impact of millets on thyroid function. Six studies (20–22, 24–26) were conducted in Sudan, one (27) in Nigeria, one experimental study (23) from Senegal studied pearl millet, and one from Guinea used fonio millet (28). Eight studies (20–27) were conducted on pearl millet and only one (28) on fonio millet. Only three of the nine studies (20, 21, 25) were on humans; the others were on rats, porcine slices, and goats.

**Table 1:** Summary of studies on goitrogenic properties of millets.

S. No	Author and year	Location	Type of millet	Study sample	Methodology	Sample size	Results
1	Osman and Fatah (1981) (20)	Sudan	Pearl millet (*Pennisteum typhoides*)	Children and households	Cross-sectional Anthropometry and goiter survey in children 72- h recall household dietary survey	1,583 children for anthropometry and goiter survey 73 rural and 70 urban households for dietary survey	Goiter was more prevalent in rural areas where as much as 74% of dietary energy is derived from millets, compared to 37% in the urban areas.
2	Osman et al. (1983) (21)	Sudan	Pearl millet (*Pennisteum typhoides*)	School girls (8–9 years old)	Mixed method included a cross-sectional survey and experimental analysis of millet content	34 school girls	1. Thiocyanate concentration was significantly elevated and that of thyroxine significantly lower in girls with goiter grades 1, 2, or 3 compared to those with grade 0. 2. A thionamide-like substance was isolated in the millets meal and suspected to be a goitrogenic factor present in millets.
3	Osman (1981) (22)	Sudan	Pearl millet (*Pennisteum typhoides*)	Rats	Experimental (in vivo)	Five rats each in the experimental and control groups	1. Feeding millets to rats produced histological changes in their thyroid gland and distorted the thyroid hormone pattern 2. The response of the thyroid gland to a millet diet depends on the duration of the experimental diet.
4	Gaitan et al. (1989) (23)	Pearl millet grain from Dakar, Senegal	Pearl millet [*Pennisetum millet* (L.) Leeke]	Female Sprague–Dawley rats, and porcine thyroid slices	Experimental (*in vivo* in rats and *in vitro* in porcine thyroid slices)	Group of four rats for experimental and control diets	1*. In vivo* feeding of bran fraction for 30 days led to a significant increase in the weight of thyroid gland of rats. 2. A marked (~85%; *P* < 0.05) inhibition of TPO was observed in the case of extracts of bran fraction in porcine thyroid slices. 3. C-glycosylflavones are active antithyroid agents in millet. Major C-glycosylflavones (glucosylvitexin, glucosylorientin and vitexin) showed antithyroid activity in the porcine thyroid slice system.
5	Elnour et al. (1997) (24)	Sudan	Pearl millet (*Permisefum americanum* L. Lecke) cultivars: 1. Balady 2. Bayoda	Male Sprague–Dawley rats	Experimental (*in vivo*)	5–8 rats in each group of experimental and control diets	Two millet cultivars (Balady and Bayoda) affected thyroid function in different ways. Only Balady cultivar was associated with significant enlargement of the thyroid gland (*p* < 0.05).
6	Elnour et al. (2000) (25)	Sudan	Pearl millet	Pre-school children	Cross-sectional survey	984 children for anthropometry 191 children for urinary iodine excretion	Supplementation of millet diets with iodine to dietary requirement did not compensate for the goitrogenic effect of millet in rats.
7	Abdel Gadir and Adam (2007) (26)	Sudan	Pearl millet (*Pennisetum typhoides*)	Male goat	Experimental (*in vivo*)	12 animals (3 animals in each group)	Pearl millet incorporated in the diet at 1 g/kg/day caused goiter in male goat kids and incorporating potassium iodate at 50 ppm did not protect animals against pearl millet goitrogenesis.
8	Goldie et al. (2014) (27)	Nigeria	Pearl millet (*Pennisetum americanum*)	Rats	Experimental (*in vivo*)	60 rats	Histopathological analysis of the thyroid gland revealed hyperplasia in rats with a 100% millet diet, while those on 60 and 30% millet diets were less affected.
9	Sartelet et al. (1996) (28)	Fonio millet sample from Guinea	Fonio millet (*Digitaria exilis*)	Porcine slices	Experimental (*in vitro*)	---	Two flavonoids (apigenin and luteolin) extracted from fonio millet caused thyroid dysfunction by altering both the TPO and the cyclic nucleotide phosphodiesterase systems.

### 3.2 Goitrogenic characteristics of pearl millet

After reviewing the published evidence, the studies were categorized into three themes: those that described the effect of pearl millet consumption on thyroid function; those that attempted to identify the goitrogenic compounds in pearl millet and those that described the effect of processing on goitrogenic potentials of pearl millet. The results of the studies included in each category are discussed below.

#### 3.2.1 Effect of millet consumption on thyroid function

The speculation that pearl millet consumption may be related to high goiter prevalence started when Osman and Fatah (20) observed that goiter was more prevalent in the villages of Western Sudan, where as much as 74% of dietary energy was derived from pearl millet, than in the urban areas, where pearl millet provided only 37% of the calories. These authors stated that the high endemicity of goiter in the region might be due to multifactorial causes rather than just iodine deficiency, and that high consumption of pearl millet could be aggravating the condition. Following this observation, Osman et al. (21) examined serum samples from girls with goiter grades 0, 1, 2 or 3 from the same region and assessed their thiocyanate and thyroxine concentrations. The results indicated that the concentration of thiocyanates was significantly elevated and the concentration of thyroxine was significantly lower in girls with goiter grades 1, 2 or 3 compared to those with grade 0. The concentrations of thiocynate in the girls with goiter grade 0, I, II/III was 9.9 ± 0.17, 11.7 ± 0.71, 12.4 ± 0.79 μmol/100 mL respectively, and the concentration of thyroxine was 8.6 ± 0.44, 6.3 ± 0.54, 7.4 ± 0.31 μg/100 mL in the girls with goiter grade 0, I, II/III, respectively. Thiocyanate at high concentrations >10 μmol/ 100 mL inhibits the incorporation of iodide into thyroglobulin by competing with iodide at the thyroid peroxidase (TPO) level, the main effect of thiocyanate is to worsen iodine deficiency (29, 30).

The authors also detected a thionamide-like substance from pearl millet meal. They suggested that the high blood concentrations of isothiocyanate observed in Sudanese schoolgirls were due to ingestion of thioamide present in pearl millet. Osman and coworkers (20, 21) thus proposed that pearl millet could be a factor in causing endemic goiter in Western Sudan.

Following these important observations on the human population of Western Sudan, Osman (22) further studied the effects of pearl millet-based diets on thyroid size and histologic structure in young male rats. The results of this study indicated that feeding pearl millet to rats produced histological changes in the thyroid gland and distorted the thyroid hormone’s production. In his experiments, the thyroid gland of pearl millet-fed rats were found to be significantly larger and heavier (*p* < 0.02) than those in the control group, with a mean weight of 58.5 mg compared to 24.7 mg, respectively. The epithelial cells of the thyroid glands of pearl millet-fed rats showed hyperplasia, or accumulation of colloid. On the basis of findings from these three studies, the authors concluded that the iodine deficiency reported in Western Sudan was not the only factor responsible for the high endemicity of goiter; rather, a goitrogenic factor from pearl millet also could be acting synergistically with iodine deficiency.

Later, Gaitan et al. (23) investigated the goitrogenic and antithyroid effects of pearl millet diets *in vivo* in rats as well as thyroid peroxidase activity *in vivo* using porcine thyroid slices. The finding from his experiment was that pearl millet diets produce effects akin to those caused by low doses of the anti-thyroid drug methimazole. A pronounced anti-thyroid effect and a significant increase in thyroid weight along with maximum inhibition of thyroid peroxidase (TPO) activity were seen in rats fed millet bran fractions. While Gaitan et al. (23) demonstrated a significant enlargement of the thyroid gland in female rats fed pearl millet bran fractions but not in those fed whole pearl millet, Elnour et al. (24) reported different results, i.e., significant enlargement of the thyroid gland in rats fed whole pearl millet. The authors observed a rise in serum thyrotropin despite normal or even slightly elevated serum T_3_ and T_4_ concentrations in rats when fed whole pearl millet. They also reported that different cultivars of pearl millet have different effects on the thyroid gland. Of the two cultivars used in their experiment, one, the slightly dark Balady cultivar., was associated with significant enlargement of the thyroid gland (*p* < 0.05). While Osman et al. (20) found that the high endemicity of goiter in Western Sudan was mainly because of iodine deficiency and the goitrogenic factor present in pearl millet act synergistically with iodine deficiency, Elnour et al. (25) reported that pearl millet diets are goitrogenic even in the presence of sufficient iodine intake. In their experiment, supplementation of pearl millet diets with iodine to meet dietary requirements did not compensate for the goitrogenic effect of millets in rats. Elnour et al. (25) studied the association of pearl millet consumption with the thyroid hormone system in preschool children in the southern Blue Nile area of Sudan. The median urinary iodine concentration (0.79 micro mol/L) found in the subjects indicated an adequate iodine supply. The mean urinary thiocyanate concentration in the population studied was high (257 micro mol/L). As the data showed no evidence of iodine deficiency in the subjects, the authors surmised that the goitrogenic substances present in pearl millet could be instrumental in the etiology of endemic goiter in the region.

In line with the findings of Elnour et al. (25), Abdel Gadir and Adam (26) reported that pearl millet incorporated in the diet at 1 g/kg/day caused goiter in male goat kids in Nubian region of Egypt and incorporating potassium iodate at 50 parts per million (ppm) did not protect animals against pearl millet goitrogens. In another animal study in Nigeria (27), hyperplasia in the thyroid gland was reported in rats fed a 100% pearl millet diet, while the thyroid glands of rats on 60 and 30% pearl millet diets were less affected. The results showed no induction of goiter by pearl millet but there was clear evidence of hypothyroidism as shown by elevated serum TSH and decreased serum T_4_ levels.

#### 3.2.2 Goitrogenic compounds in pearl millet

Since many sulfur-containing or phenolic compounds had been identified as goitrogenic substances in other foods, Osman et al. (21), suspected that the goitrogenic element in pearl millet could be thiocyanate. Thiocyanate, have a molecular size corresponding to that of iodide, inhibits the incorporation of iodide into thyroglobulin by competing with iodide. It inhibits iodide oxidation, i.e., conversion of I^−^ to *I*_2_ by inhibiting TPO activity (31).

They also found a significantly high concentration of serum thiocyanate in girls with goiter. Osman et al. (21), examined pearl millet available in the local market for the presence of isothiocyanate. Although no cyanide-containing compounds were detected in the millet meal that is fed to the girls with goiter, a thionamide-like substance was isolated. The authors thus suggested that goitrogenic thionamide present in pearl millet could be a factor in causing endemic goiter in Western Sudan. The thionamide are thiourea-based (thionamides) compounds, their major action is to inhibit the organification of iodine and coupling of iodotyrosines, thus blocking the synthesis of hormones (32).

**Figure 2:**
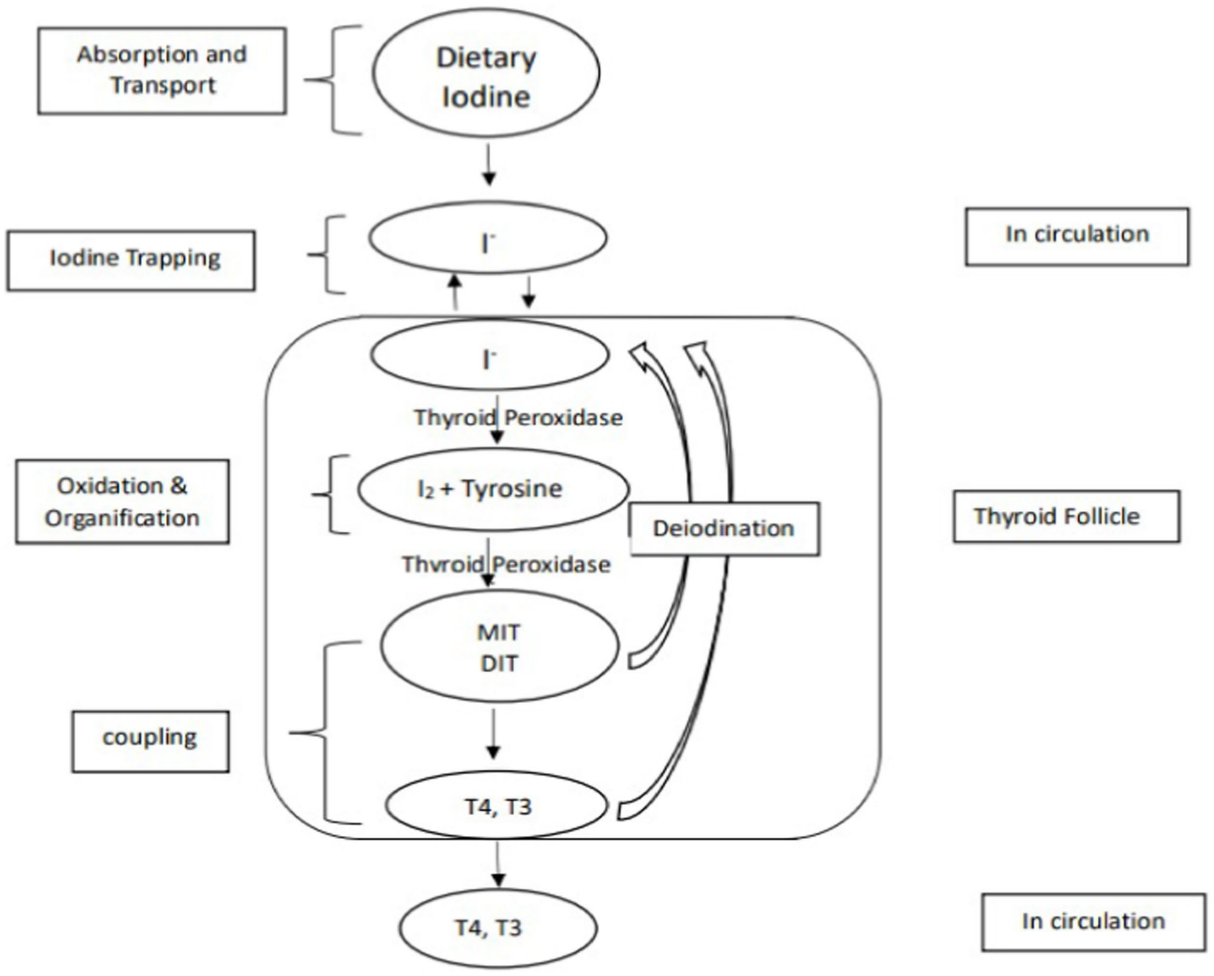
Mechanism of production of thyroid hormones.

**Figure 3:**
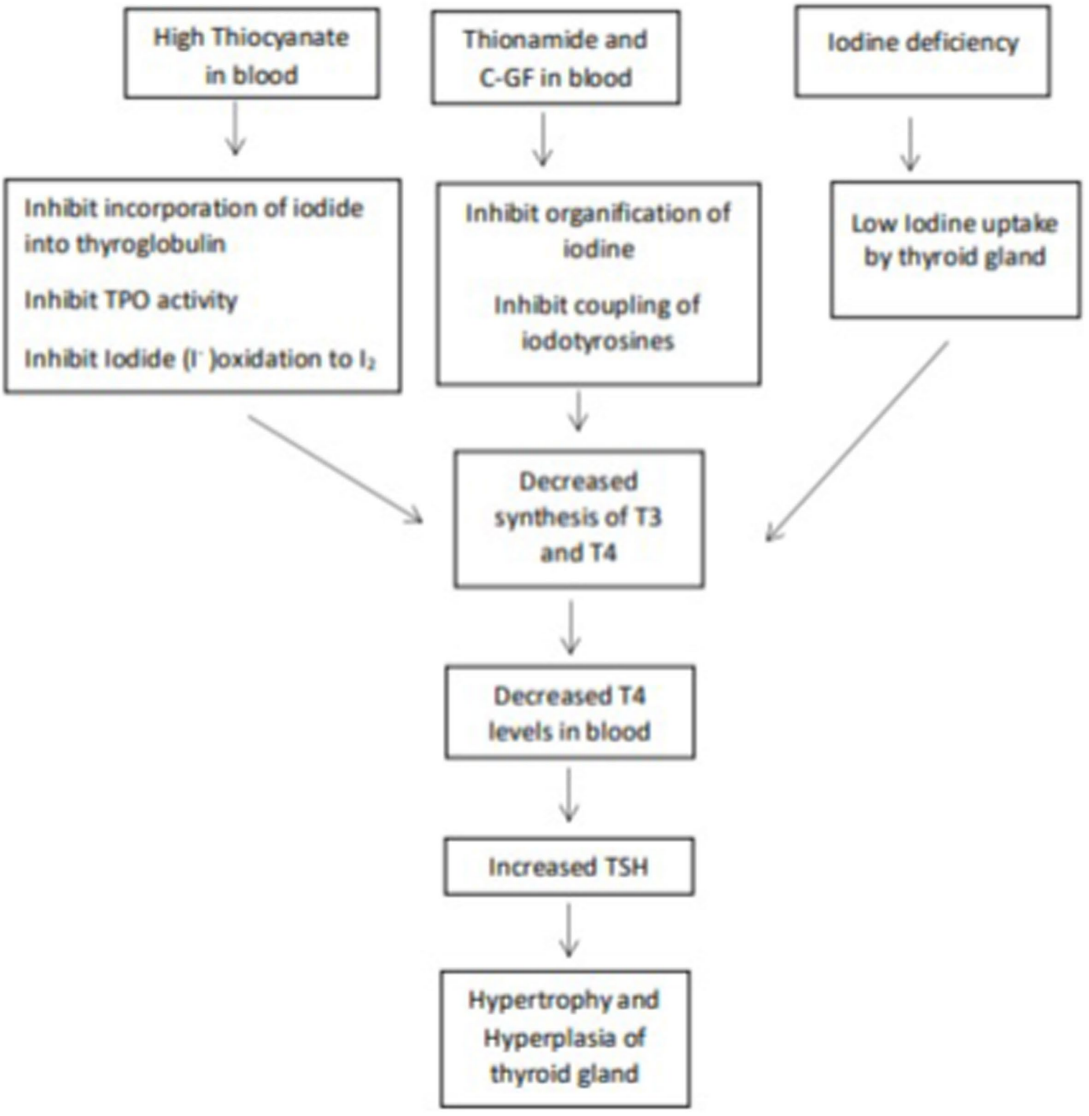
Effect of Thiocyanate, Thionamide, CGFs on thyroid gland.

Nevertheless, the quantities of thiocyanate ingested solely through different pearl millet fractions would likely be inadequate to induce goitrogenic effects (23), additionally, the goitrogenic effects of pearl millet could not be prevented with iodine supplements (24); this ruled out the suspicion that the goitrogenic element in pearl millet was a thiocyanate or isothiocyanate. The focus then shifted to the number of phenolic compounds present in pearl millet. *C*-Glycosylflavonoids (C-GFs), apigenin and luteolin are plant flavonoids and are reported as potent inhibitors of the type I iodothyronine-deiodinase, a key enzyme of thyroid hormone metabolism (33). C-GFs have been reported to inhibits TPO activities as a result the organification of thyroid iodide (incorporation of iodine into thyroglobulin for the production of thyroid hormone) is inhibited. C-GF also inhibits coupling reactions (TPO combines iodinated tyrosine residues to make triiodothyronine (T3) and tetraiodothyronine (T4). MIT and DIT join to form T3, and two DIT molecules form T4) (34). Figures 2, 3 explain the process of thyroid hormone synthesis and key steps where synthesis is inhibited by thionamide, thiocyanate or *C*-Glycosylflavonoids.

Gaitan et al. (23) examined the antithyroid activity of C-glycosylflavones (C-GF), which are concentrated in the bran fraction of pearl millet. In this study, major C-glycosyl flavones–glycosylvitexin, vitexin, and glycosylorientin–showed antithyroid activity in the porcine thyroid slice system and proved to be inhibitors of the enzyme TPO. The authors reported that C-glycosylflavones could inhibit up to 85% of TPO and concluded that they are the active antithyroid agents in pearl millet. Sartelet et al. (28) extracted two flavonoids, apigenin (150 mg/kg) and luteolin (350 mg/kg), from fonio millet. Both apigenin and luteolin manifest strong anti- TPO activities, resulting in a significant reduction in the hormonogenic capacity of this enzyme. The results of this study indicate that the goitrogenic compound found in fonio millet displays clearcut differences with that found in two varieties of pearl millet [*Pennisetum millet* (L.) Leeke and *Pennisetum americanum*] that are commonly consumed in Africa.

#### 3.2.3 The goitrogenic potential of processed pearl millet

Only two studies (23, 24) in the review investigated the effect of common processing techniques on the goitrogenic properties of pearl millet. The first, a study by Gaitan et al. (23), tested the anti-TPO activity of extracts of pearl millet bran fraction after it had been boiled and allowed to stand for as long as 2 weeks. Anti-TPO activity increased severalfold after 1–4 h of boiling and still further when the boiled extract was allowed to stand for 1 week. No further increase was observed following a second week of standing. The authors hypothesized that the increased goitrogenicity of boiled millets could be due to structural changes in the original organic constituents of millets.

The second study by Elnour et al. (24) showed that traditional fermentation increased the goitrogenic effect of pearl millet (*Pennisetum americanum* L. Lecke) cultivars (Balady and Bayoda). Rats that received fermented millet had increased serum T_4_, T_3_ and TSH compared to those that received unfermented millet.

## 4 Discussion

Pearl millet is rich in C-glycosylflavones, which is a common phenolic flavonoid-type compound and is suspected to have goitrogenic potential. The major C-glycosylflavones identified in pearl millet are C-glycosylvitexin, vitexin, and glycosylorientin (35). In animal studies, C-glycosylflavones have been shown to exert similar activities as methimazole, a drug that is used to treat hyperthyroidism. Methimazole reduces the levels of thyroid hormones by inhibiting hormone synthesis (36, 37); similarly, C-glycosylflavones in pearl millet have been suspected to affect thyroid hormone synthesis by inhibiting TPO activities.

In this section firstly the major research limitation in the study design of the included studies has been discussed, after this the beneficial effects of flavones on human health have been highlighted as these compounds have also specific functions in the body, and then the effect of simple processing techniques on the C-glycosylflavones content of millets have been discussed.

### 4.1 Limitations of the studies reviewed

This critical review of published evidence found that several of the studies had a deficient design, such as a small sample size (22) used only five rats each in experimental and control groups; Gaitan et al. (23) used group of four rats for experimental and control diets), and the presence of various confounding factors (other factors that lead to the goiter. With such limitations, sufficient statistical power on sample size could not have been reached in these studies. The major limitations in the research design of included studies were as follows:

#### 4.1.1 Few human studies

Of the nine studies included in the systematic review, six were conducted on animals and three on humans. Speculation on the goitrogenic effect of pearl millet, therefore, is largely derived from animal experimentation. Whether animals can be used to predict human response is a contentious issue (38, 39). Further, the three available human studies are cross-sectional surveys, i.e., observational studies providing insights into the prevalence of health outcomes and risk factors. We would need a cohort or an experimental design to understand the cause-effect relationship. Also, there is limited data in these studies on whether there was iodine deficiency in the humans studied. As a result, despite claiming that pearl millet has goitrogen, the studies are not supported fully by scientific data that it was the pearl millet specifically that was causing goiter in the endemic region.

The only proven information in these studies is that the pearl millet studied had C-glycosylflavones. It is important to note that several other crops, such as cabbage and cauliflower, have similar factors and are still widely consumed without any cause for concern.

#### 4.1.2 Lack of studies on the types and varieties of millet and the variability in their flavone content

High incidence of goiter was observed in the millet-consuming population of Sudan but not in the millet-consuming populations of Nigeria or India. This could be due to the high variability in flavone content in millets, which has been confirmed in different studies. Factors such as soil conditions, weather, growing location, use of plant growth regulators or pesticides, pathogen challenges, as well as date of harvest and storage time can all impact goitrogenic content in millets (40, 41).

Even though there is a marked variability in C-glycosylflavanol content in different cultivars and different fractions of millets, none of the studies, on humans or animals, took it into account this factor. Only Elnour et al. (24) pointed out that effect of pearl millet consumption on thyroid gland is modulated by cultivars used. Gaitan et al. (23) reported that the bran fraction contained greater amounts of C-glycosylflavanol than the endosperm. Akingbala (42) analyzed 17 millet varieties and found that 70% of the C-glycosylflavanol content was in the pericarp and germ fractions, ranging from 76.6 mg to 275.7 mg C-glycosylvitexin equivalent per 100 g sample. Similarly, Boncompagni et al. (43) detected and quantified vitexin, glucosylvitexin, and orientin in 97 pearl millet grain samples originating from Indian and West and Central African landraces. These authors reported a substantial variability in goitrogenic polyphenols (C-GFs) across all the pearl millet varieties in the study. The sum of the amounts of these three C-GFs (vitexin, glucosylvitexin, and glucosylorientin) varied from 15.29 μg/g to 541.10 μg/g of flour. The available studies did not take into account the variability factors.

#### 4.1.3 All the animal studies were conducted on raw millets or isolated fraction of millets, or millets as the only diet component

All the animal studies used only raw millets, either whole or the isolated fraction. The experimental animals were fed only millets. This does not represent a typical diet of animals or humans. It is worth noting that Goldie et al. (27) observed hyperplasia in rats on a 100% pearl millet diet, while rats that received 60 and 30% pearl millet diets were less affected. Similarly, in the study by Gaitan et al. (23), the bran fraction that contained maximum C-glucosylflavone content exhibited detrimental effects while the whole pearl millet fraction did not show any anti-thyroid activity. In the study by Osman and Fatah (20), peal millet was the main staple food of the population and provided 70% of dietary energy, indicating that the diets were predominantly based on millet and lacked diversity. In addition, it is not clear if the detrimental effects were due to goitrogenic factors present in a pure millet diet or due to imbalance of nutrients that may occur as a result of relying only on pearl millet. Therefore, it is not certain whether these compounds would be as harmful when eaten along with other foods or as part of a balanced diet as they might seem to be in isolation.

#### 4.1.4 Presence of multiple etiological factors like preexisting iodine deficiency and malnutrition

Multiple environmental and nutritional factors play important roles in thyroid dysfunction (44, 45). The study by Osman and Fatah (20), which claims an association between millet consumption and incidence of goiter, reported a marked deficit in all anthropometric measures of the surveyed children. Moreover, the study region was mentioned as being iodine deficient. The relevance of studies on goitrogens in iodine-deficient participants is debatable. A subclinical or clinical thyroid dysfunction or insufficient iodine intake might have an influence on the effects mediated by flavones and, therefore, such factors need to be analyzed and considered (46). Nutritional and environmental factors that may affect thyroid hormone synthesis and metabolism should be considered thoroughly as it may involve a complex interplay of various factors including C-glycosylflavones in millets. The study by Elnour et al. (25) conducted in Sudan, which showed that urinary iodine excretion (UIE) was normal in a population with 22% prevalence of goiter, indicates that the population probably consumed normal levels of iodine although it is not direct evidence of iodine intake and its level in the body. However, the study assumed that the goiter observed may be associated with defect in iodine metabolism in the body.

When we carefully evaluate this, it is noteworthy that the study failed to describe the details of regular food intake by the affected population. However, it says the population consumes millet as a staple. This is not adequate to understand any abnormalities in the body that is caused by the dietary factors. There is also a possibility that consumption of millets with low dietary diversity could have been the reason for defect in iodine metabolism. At this point, it is important to note that the researchers could not explain the sources of iodine in a region of severe iodine deficiency where iodized salt was not produced nor was iodine prophylaxis implemented at the time of the study. The high prevalence of protein-energy malnutrition along with multiple vitamin deficiencies in this population could potentially explain, at least in part, the derangement in thyroid hormone metabolism. It may be noted that human studies on the effects of millet consumption on thyroid functions in healthy individuals are lacking. More research that takes into account additional factors that influence thyroid function is strongly needed before arriving at a conclusion regarding the effects of millets.

### 4.2 The beneficial effect of flavones on human health

While a handful of studies have implicated flavonoids in pearl millet to cause goiter, several other studies have shown a wide range of protective health effects. Vitexin and its isomer isovitexin have been proven to be good radical scavengers, natural antioxidants (47–49), with anti-inflammatory (50, 51), anticancer (52, 53), hepatoprotective (54), cardioprotective (55, 56), and neuroprotective effects (57). The health benefits of eating flavonoids could potentially far outweigh any potential negative nutritional effects.

### 4.3 Effect of processing on C-GFs content in millets

Millet grains are generally dehulled before being processed for human consumption (43). Apart from dehulling, milling is done to separate the bran, germ, and endosperm portions. As C-glycosylflavanols are mostly concentrated in the pericarp and germ, any process that removes these fractions, like dehulling and decortication, substantially reduces the C-glycosylflavanol content. Malting and thermal processing dramatically reduce the number of phenolics present in millets. Simple household processing techniques such as heating denature the goitrogenic compounds into metabolites that are less likely to be harmful (58). While Gaitan et al. (23) reported that antithyroid activity of millet bran increased after heating and further increased when the boiled extract was left to stand in the same boiled water, Akingbala (42) showed that cooking, decortication, and steeping decorticated grain in water for a short time and discarding the water reduces the C-glycosylflavanol content in millet flour. Thus, levels of C-glycosylflavanol in millet foods and beverages are usually considerably lower than in the raw grains. No studies have been done on how changes in C-GFs before and after processing affect the goitrogenic content of millets.

Based on available evidence, it can be summed up that pearl millet may exhibit goitrogenic properties in concentrated, isolated or raw form. After processing and cooking and consuming a diversified and balanced diet, any possible deleterious effects can be substantially reduced.

## 5 Conclusion

Millets have demonstrated important nutritive and health properties. Existing evidence to support the claim that pearl millet consumption causes goiter does not stand up to scientific rigor. This is so for several reasons: no control groups and intervention groups were fed with the treatment diet (pearl millet or non-millet) in any of the human studies; there was no clear proven cause-effect, and the role of variables such as iodine deficiency was not clarified substantially; very wide variations in flavanols in pearl millet were not taken into account; studies were not undertaken on healthy humans; and all the animal studies used only raw millet and a 100% millet diet, which is not realistic. However, some suggestive evidence of a potential influence on thyroid function justifies the need to undertake population-level and clinical studies on the effects of millet consumption on inducing goiter formation. Research on this aspect is currently limited to three human studies involving only pearl millet and done *in vitro* or *ex vivo* but not *in vivo*. Well-designed human studies are required. More scientific research is warranted on the goitrogenic effects of pearl millet and other small millets and to understand any underlying mechanisms at work. In addition to flavones, millets provide a plethora of other nutrients and phytochemicals, and consuming millet as part of a diversified diet would not pose any risks to thyroid functioning in healthy individuals. It is recommended that diets should contain appropriate levels of iodine, e.g., cooking with iodized salt, to ensure the required iodine uptake to avoid thyroid problems.

In conclusion, the literature review clearly emphasized the paucity of evidence and flaws in the limited evidence available. This calls for more appropriate research to ensure more accurate information for better decision-making. This not only requires population and laboratory studies but also a value chain approach to determine the effect of various factors ranging from seed varieties and production to consumption.

## Data availability statement

The original contributions presented in the study are included in the article/supplementary material, further inquiries can be directed to the corresponding authors.

## Author contributions

SA: Funding acquisition, Writing – review & editing. SU: Writing – original draft. SG: Writing – review & editing. JK-P: Conceptualization, Funding acquisition, Supervision, Writing – review & editing.
